# Computer-Vision- and Edge-Enabled Real-Time Assistance Framework for Visually Impaired Persons with LPWAN Emergency Signaling

**DOI:** 10.3390/s25227016

**Published:** 2025-11-17

**Authors:** Ghadah Naif Alwakid, Mamoona Humayun, Zulfiqar Ahmad

**Affiliations:** 1 Department of Computer Science, College of Computer and Information Sciences, Jouf University, Sakaka 72341, Al Jouf, Saudi Arabia; 2 King Salman Center for Disability Research, Riyadh 11614, Saudi Arabia; 3 School of Computing, Engineering, and the Built Environment, University of Roehampton, London SW15 5PJ, UK; mamoona.humayun@roehampton.ac.uk; 4 Department of Computer Science and Information Technology, Hazara University, Mansehra 21300, Pakistan

**Keywords:** edge computing, real-time monitoring, computer vision, LPWAN, obstacle detection, assistive technology, wireless communication, deep learning, healthcare

## Abstract

In recent decades, various assistive technologies have emerged to support visually impaired individuals. However, there remains a gap in terms of solutions that provide efficient, universal, and real-time capabilities by combining robust object detection, robust communication, continuous data processing, and emergency signaling in dynamic environments. In many existing systems, trade-offs are made in range, latency, or reliability when applied in changing outdoor or indoor scenarios. In this study, we propose a comprehensive framework specifically tailored for visually impaired people, integrating computer vision, edge computing, and a dual-channel communication architecture including low-power wide-area network (LPWAN) technology. The system utilizes the YOLOv5 deep-learning model for the real-time detection of obstacles, paths, and assistive tools (such as the white cane) with high performance: precision 0.988, recall 0.969, and mAP 0.985. Implementation of edge-computing devices is introduced to offload computational load from central servers, enabling fast local processing and decision-making. The communications subsystem uses Wi-Fi as the primary link, while a LoRaWAN channel acts as a fail-safe emergency alert network. An IoT-based panic button is incorporated to transmit immediate location-tagged alerts, enabling rapid response by authorities or caregivers. The experimental results demonstrate the system’s low latency and reliable operations under varied real-world conditions, indicating significant potential to improve independent mobility and quality of life for visually impaired people. The proposed solution offers cost-effective and scalable architecture suitable for deployment in complex and challenging environments where real-time assistance is essential.

## 1. Introduction

LPWAN is also known as Low Power Long Range Technology and is a type of wireless communication technology intended for use in IoT applications with low data rates, long ranges, and low power consumption [1,2,3]. In contrast to typical wireless network systems, LPWAN is centered on the use of minimum power to transmit small amounts of data over large distances of up to several kilometers. This makes LPWAN perfect for battery-operated devices that need to have long operating cycles, such as sensors and trackers. The main LPWAN technologies include LoRaWAN, Sigfox, and NB-IoT, with features such as bi-directional communication, scalability, and worldwide coverage [4,5,6]. LPWAN works within licensed and unlicensed spectrum bands in order to accommodate a variety of deployment situations. Its applications include smart cities and smart farming, industrial IoT applications, IoT healthcare, and other use cases where high bandwidth is not feasible or would prove expensive. LPWAN, which is a strong, efficient, and cheap solution, is one of the major drivers of IoT adoption in different industries [7].

Edge computing is an extended computing model that integrates data processing and storage at the point of data collection in devices or local servers instead of merely in cloud systems [8,9,10]. This approach decreases latency, increases real-time processing, and decreases the usage of band width because edge devices in the network can analyze and process data without sending it to a central processor. Edge computing is most useful in scenarios where quick decisions must be made: for instance, self-driving cars, smart factories, smart cities, and smart health [11,12,13]. Since data can also be processed locally as part of edge computing, privacy and security are increased as information does not always have to reach a cloud located in a distant geographical location. Furthermore, it offers reliability in situations where there is an internet connection from time to time because most edge devices can operate with offline modes. This paradigm supports cloud computing and shifts prominent parts of computing to the edge, which makes the resulting systems more efficient, scalable, and responsive [14,15].

Computer vision has been very beneficial in assistive technology as it offers solutions in real time through detection and monitoring frameworks to the visually impaired [16,17,18,19]. Object detection algorithms, such as the YOLO and SSD models, are suitable for computer vision applications due to their high accuracy, fast speeds, and efficiency [20,21,22]. YOLOv5 has the ability to recognize visually impaired people, white canes, and obstacles in real-time environments. It is not complex and, as such, it can be deployed directly on the edge devices for the higher speeds required, especially when providing help [20,21]. By implementing YOLOv5 with wearable or portable gadgetry, blind individuals are able to navigate an environment through audio or tactile cues. In addition, when integrated with LPWAN technology, the designed framework can also broadcast real-time location and environment-specific alarms to the caregivers to improve the second layer of supervision and security. This combination of the powerful YOLOv5 and the connection-oriented LPWAN is a harmonious approach to enhancing the motion control, self-sufficiency, and safeguarding of the blind in different environments [7,23,24].

In this study, we present a novel assistance system in real time based on computer vision, edge computing, and LPWAN technologies; it integrates a framework that can help visually impaired people. Our approach differs from existing assistive systems by spatially deploying YOLOv5-based object detection on edge computing and LPWAN networks, which provides real-time processing and reaches larger communication distances. By incorporating edge computing, latency is reduced as it is able to process and make local decisions on data rather than those dependent on centralized cloud servers. In addition, we provide a dual communication model, using Wi-Fi as our primary channel and LoRaWAN for emergency signaling, where the Wi-Fi channel is used to achieve reliable communication in challenging or network-limited environments [25]. The main contributions of this research work are highlighted below:

- We propose and design a framework that provides an opportunity to immediately help the visually impaired person by utilizing LPWAN technology and the computer vision algorithm for object recognition and classification.
- We used the YOLOv5 model of deep learning for object detection to identify and isolate obstacles and paths or other guiding tools such as white canes at a very high speed.
- We integrated edge computing into the conceptual framework to support data pre-processing and analysis at the edge, since prompt decisions are vital when helping visually impaired persons.
- We used LPWAN as a cost-effective solution for extensive-range, low-power connections for location tracking, environmental conditions, or remote help in real time using LoRaWAN.

The rest of the study is organized as follows: Section 2 describes the related work. Section 3 presents the system design and model. Section 4 presents a performance evaluation, and, finally, Section 5 concludes the article by indicating several future directions.

## 2. Related Work

We reviewed the related work in the context of LPWAN technology, edge computing and computer vision methods that can be used to assist individuals with special needs.

The authors of [26] provide a detailed description of Emergency Communication Systems (ECS) scenarios and the current research approaches related to the utilization of unconventional and combined classic and unconventional approaches to set up communication between a specific site and the rest of the world. The ECS includes an independent wireless 4G and LTE base station and a LoRa using an IoT integration of LPWAN and SD (Software Defined)-WAN. The LoRa-based wireless network was modeled and analyzed using NS3, where LoRa basically means firm and sufficient data transfer between an appropriate gateway and the LP-WAN sensor nodes to assure trustworthy communications. The proposed scheme offered effective data transfer, which incurs minimal data losses through proper installation of the gateway at the premises, while the SD-WAN scheme that was modeled and tested using the MATLAB simulator and LTE Toolbox, together with an ADALM PLUTO SDR device, was an exceptional backup communication system as well. The performance was determined after reassembling all the received data blocks, resulting in a favorable recommendation for researchers and practitioners on the advantages of an on-premises IoT communication platform.

The research presented in [27] contrasts the various types of LoRa (long-range) design with low power consumption. Cloud technology is applied in accident identification and transport of emergency ambulances from the scene of the unintended event to the nearest hospital where accident health care can be offered. Similarly, emergency data can be dispatched to the cloud immediately, and the response is to alert the environmental entities and notify the right clinic. The presented model created an excellent vehicle as a framework involving GPS for identifying the accident spot and reaching the scene of the accident early and impact sensors for identifying obstacles.

The study presented in [28] offers a brief analysis of recent electronic travel aid (ETA) research prototypes that use smartphones for aided navigation and orientation in indoor as well as outdoor environments and offer further details about the objects in the vicinity. The PRISMA method was applied for a systematic analysis of scientific achievements in the field. A comparative meta-analysis demonstrated how several smartphone-based ETA prototypes could help in gaining better orientation, navigation, and wayfinding in both indoor and outdoor environments. The study highlighted the relatively low engagement of researchers in integrating haptic interfaces with computer vision for smartphone-based ETAs for the blind, few efforts to use new advanced computer vision techniques using deep neural networks, and a lack of empirical studies on existing commercial navigation aids.

The authors of [29] design a new wearable navigation system for guiding the user towards the target object of interest in unknown environments. The system contains two key visual computing functions: initial target object identification in 3D and real-time tracking of the user’s path, both using the 2D video recorded by a low-cost monocular camera placed in front of the chest of the user. These functions allow the system to propose an initial route, refine the route as the user is traveling, and provide the user with timely advice about correction of the route. From experiments, the authors proved that our system can work with an error of not more than 0.5 m in both outdoor and indoor environments. No other sensors are required for navigation, and all operations can be performed with the Jetson processor in a wearable system to provide real-time navigation assistance.

The research presented in [30] presents an exploration of the current research available on adaptive edge computing systems. The authors employ a well-recognized classification scheme that outlines the key facets of the management of adaptive behavior in computing systems. The authors also review how several aspects are addressed in the literature and present the open research challenges and the future direction in adaptive edge computing systems. The outcome of research is that, out of all the approaches, most of them address adaptation at the application level. The changes that may occur in the context, the changes that are induced by the users, or the changes that occur in the system and make it necessary to introduce certain adaptations are also less thoroughly investigated. The majority of the existing studies used reactive adaptation, while proactive adaptation is crucially needed to keep the edge computing systems’ performance and compatibility operable by predicting the necessary adaptations on the fly. Most of the methods use centralized adaptation control, which is somewhat unsuitable for the mostly decentralized/distributed edge computing environments.

The research presented in [31] offers the design of a wearable assistive system enabled by AI edge computing to guide visually impaired consumers in using marked crosswalks or zebra crossings. The wearable assistive system under development includes smart sunglasses, an intelligent waist belt, and an intelligent walking stick. Real-time zebra crossing image recognition is achieved by means of a deep learning method. When approaching a zebra crossing, the visually impaired consumers have to put on the proposed smart sunglasses and hold the proposed waist-mounted intelligent device; the consumer also has to hold the proposed intelligent walking cane. As soon as a visually impaired pedestrian reaches a zebra crossing, the system will send him or her a message about the current situation at the crossing and at the traffic light signal. The real-time recognition of zebra crossings of the proposed system is as high as 90%.

Existing studies in the literature [26,27,29,30,31] have already contributed a lot to assistive technologies for visually impaired individuals by incorporating LPWAN, edge computing, and computer vision. Most of the approaches deal with emergency communication systems and accident detection using LPWAN but do not discuss real-time navigation for visually impaired individuals. Studies of electronic travel aids (ETAs) [28] based on smartphones heavily depend on haptic feedback integration and deep learning for accurate object detection in smartphone-based ETAs. Basic tracking and guidance can be provided by wearable navigation systems; however, they are not able to integrate multigraphics sensors; hence they are less feasible in complex environments. Similar to the current adaptive edge computing solutions, most of them adapt in a reactive way, rather than in a proactive way, which serves to decrease system efficiency in dynamic real-world scenarios. Some of the AI-driven assistive systems for pedestrian guidance have been proposed, but mostly to detect crosswalks and do not go beyond broader guidance or emergency response mechanisms. In order to resolve such challenges, we developed a real-time detection and monitoring framework using YOLOv5-based object detection, edge computing for low-latency processing, and LPWAN for reliable emergency communication.

## 3. Proposed Framework

**Figure 1:**
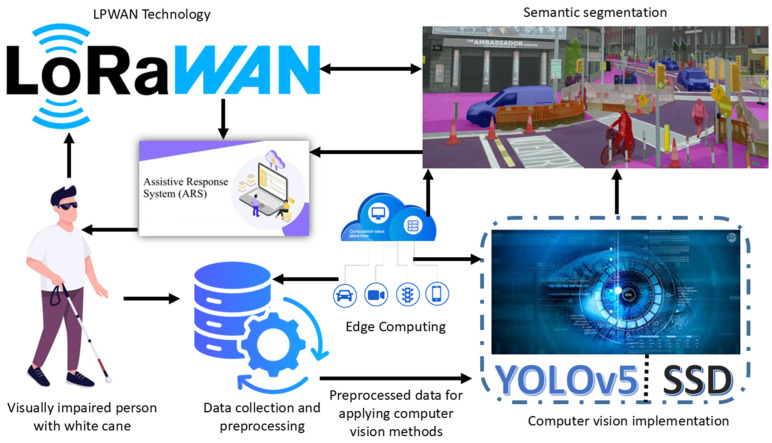
Computer-vision-based real-time detection and monitoring framework for visually impaired persons.

The proposed framework provides a solution for helping visually impaired people by implementing advanced technologies. This framework incorporates computer vision, edge computing, and LPWAN systems for improving real-time detection, monitoring, and emergency management, as shown in Figure 1. The main objective of the system is to enable it to respond as quickly as possible to the needs of the visually impaired people through the use of a reliable object recognition and communication/tracking system. The core of the system is the YOLOv5 deep learning algorithm that enables real-time object detection and recognition with fairly high accuracy to detect obstacles, paths, and guiding tools, including the white cane. Furthermore, semantic segmentation techniques are used to capture the environment and make the system more sensitive to changes in the environment. Through data processing and analysis in edge computing, the framework guarantees minimum latency responses, which are essential in cases involving the visually impaired, for instance, in real-time situations.

The incorporation of LPWAN technology adds more depth to the existing functions of the system. LPWAN is also an affordable solution for low-power, long-range connectivity and allows for real-time user location tracking, environmental sensing, and remote assistance. In particular, LoRaWAN technology was integrated as Wi-Fi backup to allow the device to continue transmitting while in regions with no Wi-Fi connectivity or during a power outage. The two-way communication system provides the system with reliability in the event of an emergency, thus improving the safety and security of the visually impaired. The hardware parts of the framework are two Heltec Wi-Fi LoRa 32 V2, the system’s IoT development boards, BLE, Wi-Fi, and LoRa. These devices work in pairs with sender–receiver functionality to ensure proper information exchange. In addition to the point, a panic button, which is one of the salient components of the system, enables visually impaired users to send distress signals within the shortest time possible. This alert sends the user’s location to a specific receiver through the LoRa network, which guarantees a quick response in cases of emergencies.

The proposed framework is considered to be the most functional and revolutionary since it employs state-of-the-art computer vision techniques, edge computing, and LPWAN. Thus, by providing real-time object identification, stable data transmission, and quick reaction to emergencies, the system also tackles the key issues of this endangered population. The study also sheds light on positive outcomes that could be achieved by applying technology to create more adaptive environments, opening up possibilities for smart cities and assistive technologies.

### Algorithms for the Proposed Framework

Algorithms 1–3 present the functioning of the core elements of the proposed framework. Algorithm 1 is based on YOLOv5 deep learning to detect and recognize objects (such as obstacles or guiding tools, including white canes) in successive video frames. A digital pre-processing of the input video frames is applied, and the frames’ dimensions are normalized and then converted into tensors. From the study, we learn that YOLOv5 detects objects by predicting a bounding box for an object and a class probability distribution. These detected objects are classified into certain defined classes (for instance, “obstacle” or “path”), and the result is given for further consideration.

Algorithm 2 is used by the edge computing device for processing detected objects in order to facilitate early decision making. On this basis, object detection data is received, object size is scaled relative to the frame size, and semantic segmentation is used to define sections of the environment. The distance of each detected obstacle to the user is determined depending on the coordinates of the user. The processed data that are useful in decision making (for example, direction finding or evasion of obstacles) are given out in real time.

Algorithm 3 effectively guarantees the delivery of emergency alerts to visually impaired individuals. In case of pressing a panic button, an alert packet includes the ID of the device, GPS coordinates, and time stamp. If Wi-Fi is unavailable, the alert is sent through the LoRaWAN network, thereby providing continuous communication in cases of no Wi-Fi or in case of an emergency. A backup alert is through Wi-Fi if available, so there is an assurance that the emergency alert message will be delivered successfully. There are two key situations where the deployment of the primary Wi-Fi was found to be unavailable. The first scenario concerns the fact that Wi-Fi coverage is often not very strong or even nonexistent in outdoor or remote areas such as parks or underground places. In such cases, the system will automatically switch to LoRaWAN for emergency alert transmission. Second, when our ISP or power outages fail, Wi-Fi routers stop working, cutting the connection. The system will automatically rely on LoRaWAN as a backup to ensure continuous communication and always obtain successful emergency alerts.
**Algorithm 1: Object Detection and Classification Using YOLOv5****Input:**   V = (blind, white cane): Image with two categories **Output:**  D: Detected objects with classifications and bounding boxes**1.** **Initialize:**Load pre-trained YOLOv5 weights W Set the input image resolution I_r_**2.** **For each frame F∈V:**Resize F to I_r_ Convert F into a tensor representation T(F)**3.** **Run object detection:**D=YOLOv5(T(F),W)where $D=\{(x_{i},y_{i},w_{i},h_{i},c_{i})\}$

$\left(x_{i},y_{i},w_{i},h_{i}\right)$ are bounding box coordinates
$c_{i}$ is the class label of object i**4.** **Classify objects:**For each detected object $c_{i}$, map the label to predefined categories (obstacle, path)**5.** **Output:** Return the set of detected objects D


**Algorithm 2: Edge Computing for Real-Time Data Processing**
**Input:**   D: Detected objects D from YOLOv5 **Output:**  D′: Processed data for decision-making**1.** **Receive data:**Transmit D to the edge device.**2.** **Pre-process data:**Normalize object dimensions:

$x^{\prime }=\frac{x_{i}}{W},\;\;\;\;y^{\prime }=\frac{y_{i}}{H}\;$

 where, W and H are the frame width and height respectively**3.** **Perform semantic segmentation:**Apply segmentation model S to identify environmental regions

M=S(F) 

where M is the segmentation mask.**4.** **Analyze obstacles:**For each obstacle $c_{i}$, calculate proximity $P_{i}$ to the user: 

$P_{i}=\sqrt{{\left(x_{i}-x_{u}\right)}^{2}+{\left(y_{i}-y_{u}\right)}^{2}\;}$

where, $\left(x_{u},\;y_{u}\right)$ are the user locations. **5.** **Output: Generate real-time decision-making data D′**


**Algorithm 3: Emergency Alert System Using LPWAN**
**Input:**   S_b_: Panic button signal, GPS Location (Lat, Lon) **Output:**  Emergency alert message**1.** **Monitor panic button:**if S_b_ = 1 (button pressed), proceed to step 2. **2.** **Generate alert:**Create alert packet P:   P={ID, Lat, Lon, Timestamp} where ID is the device ID, Lat, Lon are GPS coordinates, and Timestamp is the current time**3.** **Transmit alert:**Send P via LoRaWAN if Wi-Fi is unavailable

Transmit (P, LoRaWAN) 


**4.** 
**Backup alert via Wi-Fi:**



Transmit (P, WiFi) 


**5.** **Output:** Successfully delivery of the emergency alert


## 4. Experiments, Results and Discussion

We performed simulations and evaluated the performance of proposed framework with respect to the object detection and classification, while we performed real-world experiments with regard to the implementation of LPWAN.

### 4.1. Computer-Vision-Based Object Detection and Classification

- Dataset

The “Visually Impaired (White Cane)” [32] dataset available on Kaggle platform was used; it contains a total of 9309 image files, categorized into two main classes (blind and the white cane). This dataset is useful for the training of machine learning models that are specifically used for identifying visually impaired people and white canes in different scenes. They are appropriately tagged, which makes it easier for supervised learning problems to occur on each image. The dataset is especially suitable for initiatives aimed at improving navigation and security for those with vision impairments by using computer vision.

b. Dataset Preprocessing

We preprocess the “Visually Impaired (White Cane)” dataset to transform it into the form required by machine learning algorithms. The data are in .jpg format with .txt label files, which are then preprocessed in order to match the images and their labels. The preprocessing involves splitting the dataset into three subsets: The prevalence of the training set was 70%, while that of the validation and test sets was 20% and 10%, respectively. Organizing the data in this way allows for the equally effective training, fine tuning, and evaluation of machine learning models with subsets of data.

For each subset, we create separate directories for images and labels so that there can be no confusion. Image files are first extracted and then sub-categorized into different sets using the method train_test_split from sklearn. Then create text files with the name of each image and replace the extension with .txt and move them to the correct directories. This systematic approach means that there is always a proper correlation between images and their labels in the different subsets. We also make the dataset usable in machine learning pipelines by handling file operations autonomously.

c. Implementation of Yolov5 Model

The YOLOv5 model was used in the context of object detection of the “Visually Impaired (White Cane)” dataset. The intention was to correctly identify and locate visually impaired people and their walking sticks, or white canes, in the images. As a result, YOLOv5 was used due to its fast and accurate detection method and was able to perform real-time object detection.

The dataset is made up of images and their associated label files in which each label contains the bounding box coordinates and the class number of objects in the image. The bounding boxes are tagged in the normalized coordinates, and the tags are then converted back to the original image scale when needed. For data preparation, we read the label files, calculated the corners of the bounding boxes, and, using a script, we overlaid these bounding boxes on the images, which gives a visualization of the models predictions. Other objects were bound with the class IDs written above each box to make it easier to interpret the results given by the model. This step was useful in checking on the correspondence between the labels to the images and the quality of the YOLOv5 predictions. For the bounding boxes, a red rectangle was drawn to help in interpretation, and the class labels were written using text annotations. The applied YOLOv5 model consists of 7,012,822 parameters and 157 layers, with a computational complexity of 15.8 GFLOPs. It was trained for 24 epochs, taking 0.801 h (approximately 48 min) in total, or about 2 min per epoch. Finally, with training and optimization, the final model size was 14.4 MB, obtaining proper performance and resource balance.

**Figure 2:**
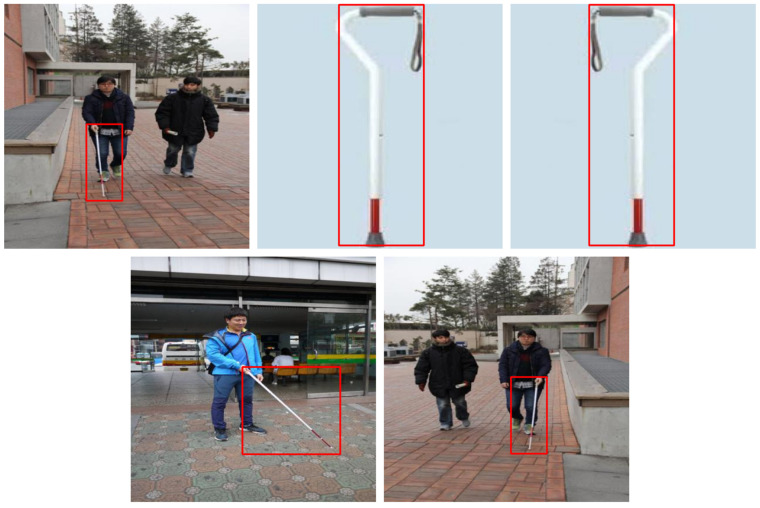
YOLOv5 model’s ability to detect objects from visually impaired (white cane) dataset.

As illustrated in Figure 2, the different visualizations confirmed the ability of YOLOv5 to identify objects with high accuracy, given that the dataset was well structured with clear labeling of objects. The process not only showed the strong sides of the model but also regions where perhaps false positive or false negative results can occur. These visual checks coalesced with the quantitative measures collected during the model assessment phase and highlighted areas to improve within training.

We also remove the optimizer state from the saved model weights file (last.pt and best.pt) to make it compressed and to improve the speed of the model during inference. We test the model with the best weights (best.pt) on a dataset of 1871 images; 1909 instances were detected. We combine the layers of the proposed model to fine tune it from the perspective of faster inference. Next, we briefly describe the model architecture with 157 layers and 7,012,822 parameters that takes 15.8 GFLOPs to pass forward. Then, we evaluate the Yolov5 model performance and obtain key metrics, which are presented in Table 1. The metrics in Table 1 indicate strong performance in both detection and classification tasks, with the model achieving high precision and recall.

**Figure 3:**
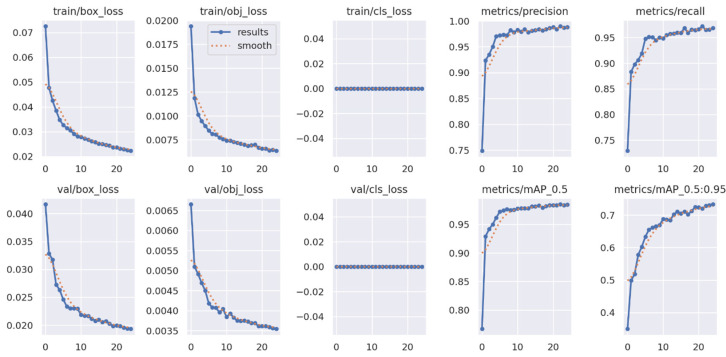
Loss metrics with precision and recall curves.

We then plot the results using a plot shown in Figure 3, which shows the various loss metrics, which include box_loss, object_loss, and class_loss, as well as the precision and recall. These metrics indicate how the learning process is going in the course of model training; that is, they indicate how the model’s abilities to predict the coordinates of bounding boxes, to detect objects, and to classify them correctly are changing. The plot also contains the precision and recall statistics that show how well a model was able to find objects (precision) and did not miss most of them (recall). The high values of precision and relatively high recall value mean that the model has very small values of both false positives and false negatives; hence the model would work well practically. Therefore, we can safely state that the YOLOv5 model is efficient and ready for deployment or additional fine-tuning.

d. Implementation of SSD Model

We carry out exploration analysis on a dataset and then perform an image detection using a Single Shot MultiBox Detector (SSD) model. The objects to be detected in this particular model include, for instance, visually impaired people and white canes, and the performance of the model is assessed using the validation set. To measure the models’ performance on the problem, we compute various parameters including precision, recall, and mean average precision (mAP) with IoU thresholds that define successful prediction. While evaluating, prediction is calculated for each image present in the validation set. Such predictions are based on the presence of bounding boxes and confidence scores and are compared to the ground truth boxes via IoU. Detection results that have a higher IoU of more than a certain level (for example, 0.5) are considered correct while the rest are labelled as false positives. Additionally, the ones that are not matched with the corresponding prediction are considered false negatives among ground truths. Using these outcomes pooled over the dataset, we calculate precision, recall, and mean average precision as one composite measure.

The measures obtained from the analysis are precision of 0.0023, recall of 0.6634, and mAP of 0.3328 (Table 2). These scores mean that, although the model has a high recall rate, meaning that most objects are identified, it may suffer from low precision, apparently because it often produces false positives. These parts of the performance suggest that a focus on improving the model structure, changing the anchors, or adding more data to the training set will increase accuracy.

The SSD model was implemented for detecting visually impaired individuals and white canes, and its performance was not satisfactory with a precision of 0.0023, recall of 0.6634, and mAP of 0.3328. But the detection accuracy on the high false positive rate is not satisfactory, so we chose to transfer to the YOLOv5 model that worked much better. The most accurate YOLOv5 attained a precision of 0.988, recall of 0.969, and mAP of 0.985, which is the highest compared to the other methods. With these improvements, YOLOv5 was chosen as the model of choice for our framework.

e. Implementation of Semantic Segmentation

We perform semantic segmentation for the assistive navigation systems to obtain pixel-level information about the image for object detection and mapping of the environment. When performing this, we employ DeepLabV3+, a segmentation model that has shown high levels of performance when it comes to semantic segmentation. The task is thus to map the existing annotations of object detection to segmentation masks and subsequently train the model to output a pixel-wise label for each object in the image.

We address the issue of conversion of YOLO-style annotations to segmentation masks. This is conducted for training, validation, and test datasets and uses label files that contain original annotations in the form of bounding boxes. For every box, the script converts YOLO format coordinates relative to the image size and generates a binary mask for each object using a different class number (mask color). The outcome of the mask is stored in a .png file on the image’s name. This preprocessing step readies the data for training of the DeepLabV3+ model, which in turn will segment the objects on the images at the pixel level.

Finally, based on the analysis of the dataset preparation for the semantic segmentation, it is found that the training set has 6515 images and 6515 masks, and no mask or image is lost, which shows that the training data are perfectly prepared for the training of the model. Likewise, there are 1871 images and 1871 masks in the validation set, and no data are missing in the images or the masks, which means that the validation phase will be conducted on the correct data. For the test set, there are 922 images and 922 masks with no missing images or masks; thus, they are prepared for the evaluation of the model trained in this study. The good thing is that there are no missing data in all splits, which makes the dataset complete, and we can now go ahead and train the semantic segmentation model without worrying about data-related problems.

**Figure 4:**
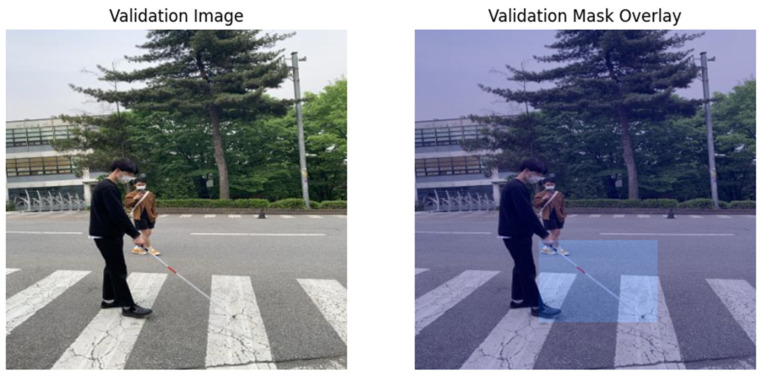
The original validation image and the overlay of the segmentation mask from visually impaired (white cane) dataset.

Specifically, we choose an image from the validation subset randomly and then show how it looks together with the segmentation mask. Originally, the image is read from the validation set directory, and the corresponding mask is obtained and reshaped to the image size. The mask is then color-mapped to improve the visualization. Originally, there is an image where the authors place the mask by using a blend of the weighted sum in a way that accentuates the regions where the segmentation model identifies the objects. Figure 4 also contains two subplots: the validation image is shown as the first subplot, and the second subplot contains an overlay of the segmentation mask that can be easily compared with the image. By using this visualization, one can evaluate the model’s performance of correctly distinguishing and grouping objects in the image.

**Figure 5:**
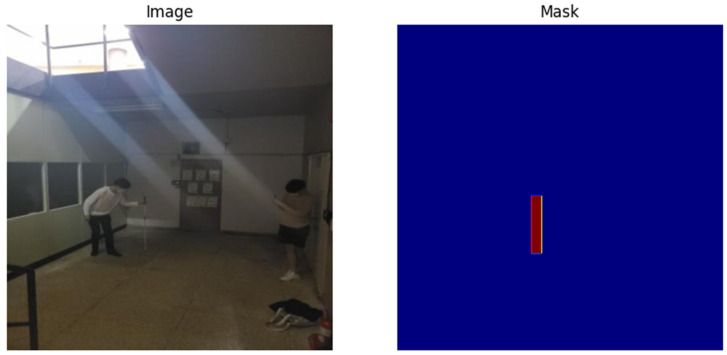
A sample image and its corresponding mask from visually impaired (white cane) dataset.

In Figure 5, we present an example of an image from the training dataset and its mask. The script now chooses a batch at random from the data loader and takes an image–mask pair out. The image is then reshaped to match the visualization height, width, and number of channels. The mask is in 2D form, and the figure is represented using a color map for enhanced visibility. What is presented on the left is the original image, and on the right one is the mask, where white color indicates the areas containing the segmented objects. This visualization allows one to review the structure and labeling of objects in the image by the model, which gives the opportunity to check the adequacy and quality of the dataset loader when preparing the data for training.

In the next step, we apply the evaluation of the model on the validation set to check how accurate the latter is. The evaluation process involves calculating two key metrics: Mean Intersection over Union (IoU) and Mean Pixel Accuracy. The model is transitioned into evaluation mode, and for every batch of images in the validation data loader, we feed the images through the model and compute the predicted outputs. We then compute IoU by identifying the intersection and union of the predicted and true masks and pixel accuracy by comparing the predicted labels with the true labels at the pixel level. The script sums up the total of IoU and the total of pixels that are accurately segmented for all the batches, and only the average values are then displayed. The validation set has been used to measure the mean IoU at 0.8320 and the mean pixel accuracy at 0.9917. The results presented here show that the model is effective in terms of both pixel-level accuracy and segmentation quality and can indeed correctly detect objects in the validation images and generate precise segmentation maps. From the quantitative results, we can see that the pixel accuracy is very high. This means that the model is right for most of the pixels, and the IoU score means the degree of overlap of the segmented object predicted by the model and the actual object boundary, which means the model is precise enough that it can be used in real-life applications where segmentation is needed.

**Figure 6:**
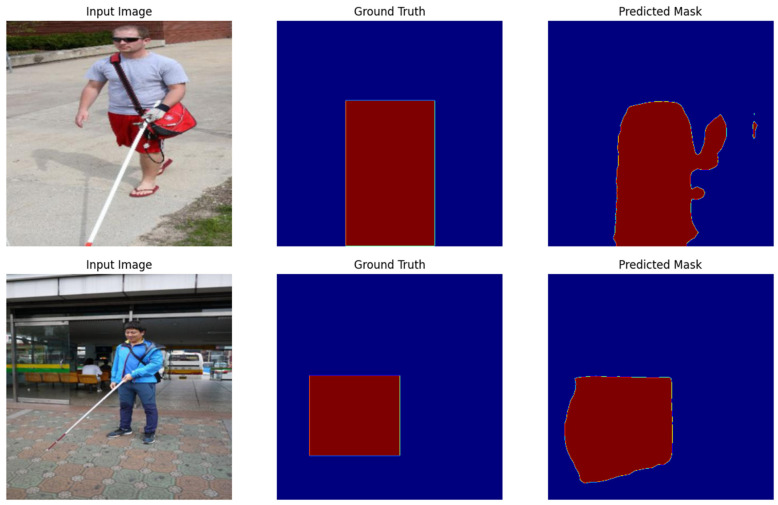
Predictions on the validation set, comparing the input images, the ground truth masks, and the predicted masks from visually impaired (white cane) dataset.

In Figure 6, we show the results of the model on the validation set, in terms of input image, ground truth mask, and predicted mask for some samples. It then produces a set of images and their ground truth masks from the validation data loader; then, it feeds them to the model to get the prediction. Coming out of the model is a set of logits, and, by applying the ‘argmax’ function, the class labels are obtained. Finally, the original images, the ground truth mask, and the predicted masks are shown in the same panel for the sake of clearer comparison.

The first inset plot in Figure 6 shows the input image, which gives a view of the original data. The second subplot is the ground truth mask, which shows the actual segmentation of the objects in the image. The third subplot demonstrates the predicted mask, which is the outcome of the model, which has gone through the semantic segmentation step. This allows us to visually judge the segmentation performance of the model, where the closer the predicted mask to the ground truth mask, the better the performance of the model. The figure shows how well the model can split objects and gives some idea of the areas where the model may require fine tuning.

To test the model’s ability to generalize to unseen data, we perform the measurement on the test dataset following training using the validation data. The evaluation process includes quantification of mean IoU and mean pixel accuracy, which are the fundamental measures in semantic segmentation problems. In the evaluation, the model is set to evaluation mode, and then an input forward pass is made on the test images. The previously described bounding boxes are used to segment the predicted masks regarding the ground truth masks to compute the IoU and pixel accuracy. The IoU is computed as the overlap between predicted and true masks as a fraction of the union of predicted and true masks; pixel accuracy measures the percentage of exactly correctly classified pixels.

The results demonstrate that the proposed model has a mean IoU of 0.8456 and mean pixel accuracy of 0.9912 for the test data set. These results suggest that the model performs very well at segmenting objects, as shown by the high pixel accuracy and a high degree of overlap between the predicted masks and the true masks (IoU). It can be inferred that most of the image pixels are correctly classified by the exceptional pixel accuracy, and the high IoU proves that the model identifies the boundaries of the objects.

### 4.2. LPWAN Implementation

We used LPWAN technology as an application for aiding visually impaired people. More specifically, the protocols we proposed and implemented were an LPWAN-based Assistive Response System (LARS). As a result, to provide efficient data distribution, we used Wi-Fi as the primary means of communication while using LoRaWAN as an additional solution where Wi-Fi connection is not available. This double layer guarantees smooth signal transmission and reception in case of connection with Wi-Fi networks problems or power outages during emergencies. LoRaWAN thus continues to operate during such interruptions, hence providing the assurance of the reliability of the system.

In our system, we use two Heltec Wi-Fi LoRa 32 V2 boards that integrate BLE, Wi-Fi, and LoRa functionalities. In this activity, one device is transmitting the signal while the other device is expected to receive the same signal. For the targeted visually impaired users, the system offers the opportunity to press a panic button, which will result in the immediate sending of the alert message to the recipient. This setup minimizes latency and makes the emergency response mechanism more reliable. The Heltec Wi-Fi LoRa 32 V2 is a low-power consumption IoT development board featuring an ESP32 dual-core processor, an SX127x LoRa/Wi-Fi/BLE, a Li-Po battery charging circuit, and a built-in 0.96-inch OLED display. ESP32 has built-in support for TCP/IP and 802.11 b/g/n Wi-Fi MAC and offers Wi-Fi Direct. The SX1276 transceiver uses Semtech’s proprietary LoRa modulation scheme, enabling high sensitivity of over −148 dBm. This high sensitivity, along with immunity to in-band interference, makes the device highly useful for IoT applications.

The GPS module integrated within the system allows location identification and tracking with at least 2.5 m positional accuracy in two dimensions. This small and inexpensive module is used for such purposes as satellite navigation, speed and location tracking, and navigation of ground, aerial, and marine vehicles. It has an IPX interface for active antenna connectivity and has low power consumption, low cost, and small size, making it ideal for portable devices. The IoT panic button is an essential part of our system that has been developed to respond to the needs of visually impaired people in emergency situations. This makes it easy to use and efficient, and users do not need to possess technical skill in order to use the application. They can push a button that would send an alert with the user’s location to the desired recipient using the LoRa network. This guarantees quick action in case of an emergency such as difficulty in navigation or cases of theft. The portability of the panic button increases its usability as well as ease of use to provide a realistic and strong solution in real-time help and monitoring in times of emergencies.

A total of 30 trials were performed to evaluate the efficiency of the system, 15 in a typical open area and the other 15 in a typical closed area. The open field was chosen because the terrain of the field is higher and steeper so as to ascertain whether the system can perform optimally in such conditions. The experiments involved using two antenna heights, 1 m and 2 m, and two packet sizes, 32 bytes and 64 bytes, with the transmission power (TX power) at 15 dBm for all tests. The end device was tested at distances of 0 m, 50 m, 100 m, 200 m, 300 m, 400 m, and 500 m. In every configuration, the experiment was carried out methodically. First the antenna was used at 1 m for transmitting 32-byte data packets and then at 2 m with the same size packet. This process was then repeated using a data packet size of 64 bytes. In each distance, fifty packets were sent, and average latency was determined. Latency is usually the time that has elapsed from a certain event, and it is relevant in the network because of the time that data takes to travel from one node to another. The transmission and reception delay time is rounded. Trip delay is commonly measured in microseconds; this period accounts for signal transmission and back. The latency is arrived at by adding several other types of delays, such as transmitting and receiving delays. It is measured in ms [33]. Equation (1) is used to compute the latency:

(1) $$Latency={Received}_{T}-{Send}_{T}$$

Table 3 shows the latency and RSSI (Received Signal Strength Indicator) with variable packet sizes and antenna heights. The performance of the system is described in the experimental results in terms of latency and received signal strength indicator (RSSI) at different packet sizes, heights of the antennas, and distances. The latency, which measures the time taken for data transmission and reception, rose uniformly with distance for all the configurations. At a distance of 1 m and a packet size of 32 bytes, latency varied between 225 ms at 0 m and 350 ms at 500 m, and, when the antenna height was increased to 2 m, the latency was between 210 ms and 330 ms, respectively. Likewise, for 64-byte packets, the latency was much higher, ranging from 745 ms at 0 m to 806 ms at 500 m for 1 m height, and it was even higher at 2 m height, ranging from 565 ms to 713 ms over the same distances. These results also show that increased packet size results in increased latency, while increasing the height of the antenna decreases the latency through improving the quality of the signal.

Similarly, received signal strength indication (RSSI) values were dependent on the packet size, height of the antenna, and distance. In the case of 32-byte packets, the RSSI values were higher (nearer 0 dBm) at short distances from the transmitter node and fell gradually with the increase in distance from the transmitter node. At 1 m antenna height, the average value of RSSI dropped from −24.82 dBm at 0 m distance to −111.15 dBm at 500 m distance. Likewise, at 2 m, RSSI reduced from −52.15 dBm to −116.12 dBm for the same distances. The pattern was similar with RSSIs being lower at 64 bytes, with overall readings starting from −56.3 dBm at 0 m and decreasing to −126.75 dBm at 500 m, with an antenna height of 1 m. An increase of the antenna height to 2 m had a positive effect on the RSSI values, which varied from −57.31 dBm for 0 m to −112.38 dBm for 500 m.

The results reveal that packet size, the height of antennas, and distance define system performance, and the need for choosing an optimal value was stated. These include reduced packet size and increased antenna height, and these are more desirable wherever there is a need to make rapid communications that are very dependable and the RSSI is strong. However, larger packets have higher latency and a weaker signal strength, an issue that can be very sensitive to changes in system parameters depending on application and environment. They also corroborate the anticipated decline in performance with distance to the target and therefore support optimal designs for the position of the antenna and strength of the signal to ensure dependable performance of the system over large distances.

We chose a 32-byte and 64-byte packet size to optimize latency, signal strength, and transmission efficiency for emergency communication in our LPWAN-based Assistive Response System (LARS). We observed that latency and RSSI values were lower when the size of the packet was smaller (32 bytes), and they were suitable for time-sensitive alerts, while introducing more delay, but this can increase data transmission size (64 bytes). Previous studies [3,7] have also considered similar packet sizes in LoRaWAN and LPWAN communication systems due to the need to balance network performance, energy efficiency, and real-time responsiveness for assistive technologies. In the experimental analysis of LPWAN implementation, we observe that an RSSI value closer to zero was obtained under cold or rainy weather conditions than under normal or hot weather conditions. In addition, low-light conditions, high traffic density, and noisy environments have been considered factors. The object detection accuracy may need optimized sensor configuration for low light conditions. The density of traffic that can generate congestion can severely dampen real-time processing and making decisions.

Our system achieves significant improvements in terms of performance. It is the YOLOv5 model that has high accuracy on object detection so that obstacles, pathways, and guiding tools can be identified. We apply the framework in our experimental results and show that it has real-time applicability by reducing latency and enhancing signal strength under varying environmental conditions. The edge computing paradigm improves decision-making speed, and thus the system becomes more sensitive to the needs of visually impaired users. From a cost-effectiveness point of view, LPWAN technology (LoRaWAN) features long-range, low-power communications, which makes it a cost-effective option as compared to traditional wireless networks. The use of edge computing reduces the need for high-bandwidth cloud processing and overall reduces operational costs as well as improving system efficiency. At the same time, the system makes use of existing IoT infrastructure such as wearable or portable devices and does not require high-powered specialized hardware.

## 5. Conclusions and Future Work

This study proposed a framework that integrates computer vision, edge computing, and LPWAN technologies to offer a more effective and real-time assistance system for the visually impaired. We used YOLOv5 for object detection; the system detects obstacles, paths, and the guiding tools in real time and with high accuracy. In edge computing, data pre-processing and decision making can be achieved locally, and the necessary help can be provided in time without the excessive use of central servers. The integration of LPWAN emergency response models, which are backed by LoRaWAN and Wi-Fi, guarantees communication in extreme situations or within complex environments. The outcomes of the experiment prove the efficiency of the framework if measured in latency and signal strength in different circumstances, which confirms its practical applicability and sturdiness. This framework can be considered an effective and low-cost solution to improving the relative independence and safety of the visually impaired people in variable environments. The proposed framework is limited to object detection capabilities, without the use of meta-learning and ensemble learning. It is also primarily for pedestrian assistance, and there is no vehicle integration into it for voice navigation. The framework is based on the existing LPWAN coverage, which may be limited in some areas, and it does not support any alternative power source such as a solar panel for improved reliability in the resource-constrained environments.

In future, we aim to develop the framework by incorporating more advanced AI models to provide a deeper analysis of objects and to improve the methods used in object detection. We also aim to extend this study with signal to provide a system that can be used in cars, i.e., a voice-based navigation aid and multi-modal interface that will be a valuable improvement. We will also consider how the LPWAN coverage can be expanded and how devices with other types of power sources, such as solar panels, can be integrated into the system for enhanced reliability and efficiency in regions with limited access to resources. We aim to extend this study by incorporating additional object classes along with diverse environmental factors and context-aware deep learning models to improve environmental understanding and system robustness.

## Figures and Tables

**Table 1:** Yolov5 model performance.

Metric	Value
Precision (P)	0.988
Recall (R)	0.969
mAP	0.985

**Table 2:** SSD model performance.

Metric	Value
Precision (P)	0.0023
Recall (R)	0.6634
mAP	0.3328

**Table 3:** Latency with variable packet sizes and antenna heights.

Packet Size	32 Bytes				64 Bytes			
Antenna Height	1 m		2 m		1 m		2 m	
Distance	Latency (MS)	RSSI	Latency (MS)	RSSI	Latency (MS)	RSSI	Latency (MS)	RSSI
0 M	225	−24.82	210	−52.15	745	−56.3	565	−57.31
100 M	248	−89.84	221	−97.44	760	−93.11	597	−86.77
200 M	269	−93.55	245	−93.40	779	−86.38	608	−94.94
300 M	278	−100.67	282	−113.15	788	−107.91	647	−106.64
400 M	291	−106.61	298	−114.86	798	−118.18	665	−121.22
500 M	350	−111.15	330	−116.12	806	−126.75	713	−112.38

## Data Availability

The “Visually Impaired (White Cane)” [32] dataset available on the Kaggle platform has been used in this study.
